# Extraction of Lignocellulose from Rice Straw and Its Carboxymethylation When Activated by Microwave Radiation

**DOI:** 10.3390/polym16223208

**Published:** 2024-11-19

**Authors:** Abdirakym Nakyp, Elena Cherezova, Yuliya Karaseva, Kaiyrzhan Shalmagambetov, Aleksandr Aleksandrov, Rakhmetulla Zhapparbergenov, Nurgali Akylbekov, Rakhymzhan Turmanov

**Affiliations:** 1 Center of Physical Chemical Methods of Research and Analysis, Al-Farabi Kazakh National University, Al-Farabi Ave. 71, Almaty 050038, Kazakhstan; kairshan@yandex.ru; 2 Institute of Polymers, Kazan National Research Technological University, 68 K. Marx Str., Kazan 420015, Russia; cherezova59@mail.ru (E.C.); karaseva_j@mail.ru (Y.K.); alexananat@gmail.com (A.A.); 3 Laboratory of Engineering Profile “Physical and Chemical Methods of Analysis”, Korkyt Ata Kyzylorda University, Aiteke bi Str., 29A, Kyzylorda 120014, Kazakhstan; ulagat-91@mail.ru; 4 “KazEcoChem” LLP, D. Konaev 12, Astana 010010, Kazakhstan; 5 Department of Science, Abai Kazakh National Pedagogical University, Dostyk Ave., Almaty 1305001, Kazakhstan; t.rahimjan.91@mail.ru

**Keywords:** rice straw, extraction of cellulose, synthesis, microwave radiation, carboxymethylcellulose, lignin

## Abstract

The paper presents the process of cellulose extraction from rice straw using water–alkaline solution treatment and the subsequent process of carboxymethylation of the obtained product when activated by microwave radiation. After mercerization of rice straw, the obtained product contained 89.2% cellulose and 6.7% lignin. The X-ray diffraction pattern of the obtained lignocellulose shows three diffraction peaks in the region typical for the polymorphic modification of cellulose Iβ (2θ = 15.50(78), 21.70(145), 34.70(52)). The degree of crystallinity was 65%. The product was heat-stable up to 247 °C. The synthesis of carboxymethylcellulose (CMC) based on the obtained product included successive processes of thermostating in alcohol–alkali solution and cellulose esterification reaction using monochloroacetic acid. To activate the carboxymethylation process, microwave radiation was used (350 W for 90 s), which made it possible to reduce the reaction time by more than 100 times. Functional group analysis of the carboxylated lignocellulose from rice straw was carried out using an FTIR spectrometer. In the IR spectra, a band with a maximum of 1742 cm^−1^ was recorded, corresponding to stretching vibrations of >C(O)OH groups. The degree of polymerization was recorded by mass spectrometry.

## 1. Introduction

Cellulose and its modification products are important components that are used to obtain valuable technical products and materials [1,2,3], including the production of paper, textiles, packaging, and others [4,5,6,7,8,9].

The main raw material source of cellulose is wood. In recent years, there has been a search for an alternative raw material base. In particular, there is an increased interest in the use of cellulose extracted from the straw of cereals, oilseeds, and bast fibrous agricultural plants [10,11,12,13,14,15,16,17,18]. This kind of raw material has a number of advantages, such as annual renewability, easy processing, and low cost.

Along with cellulose, the leaves and stems of this type of raw material contain a small amount of resins, lignin, and mineral components. Technologies for separating cellulose from the straw of various crops are similar to those for wood processing. Acid hydrolysis, an alkaline treatment followed by bleaching [19], can be used to extract cellulose from herbaceous plants to break down the outer covering tissue, which is free from mineral components, resins, and lignin. In recent years, more and more attention has been paid to the methods of extracting cellulose from annual plant raw materials based on a combination of temperature, chemical, and physical effects [20,21].

Among the main methods of cellulose modification are esterification [22], acylation [23], and carboxyalkylation [24,25]. The most widely used process is carboxymethylation. The unique properties of carboxymethylcellulose (CMC) allow it to be used in many industries, including food [26,27], pharmaceuticals [28,29], cosmetics [30,31], textiles, and petroleum [32,33,34]. CMC acts as a thickener, stabilizer, and binder, and has excellent film-forming and emulsifying properties [35]. CMC is also known as an emulsion stabilizer and is used in the development of cooling nanofluids or lubricants in cutting processes [36]. CMC is also used as a swelling filler in water–oil swelling rubbers for packaging equipment [37]. Moreover, CMC is a biodegradable polymer, making it an environmentally friendly choice.

Carboxymethylation is usually carried out using sodium hydroxide and sodium monochloroacetate or monochloroacetic acid. In the cellulose carboxymethylation process, sodium hydroxide acts as an activator, weakening the hydrogen bonds within cellulose crystallites, thereby making individual polymer chains available for uniform chemical modification. In addition, alkali increases the nucleophilicity of the cellulose hydroxyl groups [38,39], which facilitates the carboxymethylation reaction.

Suspension and solid-phase methods of cellulose carboxymethylation are described in the literature [24,40,41]. The process is carried out in aqueous or hydro-alcoholic media. However, these methods are long in duration (3–5 h) and are accompanied by the formation of large volumes of wastewater.

In [42,43], ultrasonic treatment is suggested for obtaining carboxymethylcellulose. However, this method of CMC production takes 30–40 min.

Since cellulose carboxymethylation reactions take place in polar media, there are prerequisites for using microwave radiation (MWR) to intensify this process [44]. The effect of accelerating reactions when exposed to MWR is explained by the fact that microwaves generate electromagnetic waves that provide rapid and uniform heating of the entire volume of the material. In addition, microwave radiation is thought to help break down the crystalline structure of cellulose and thereby improve effective contact between cellulose and the acid involved in the carboxymethylation reaction [45]. This makes it possible to obtain the required product in a shorter time [46,47].

The novelty of this work lies in the use of microwave activation in the process of cellulose carboxymethylation, which provides a significant reduction in the time of synthesis. The use of microwave technology can speed up the process by more than 100 times in comparison with traditional methods, which can take from 2 h to 8–10 h. This approach not only improves the efficiency of the synthesis, but also contributes to high yields of the product, making it more economically feasible. Microwave activation provides a more uniform distribution of energy in the reaction mixture, which promotes more active interaction of the reagents and increases the reaction rate.

Rice straw is used as the object of the study. This is due to the fact that rice is one of the most widespread and widely consumed cereals all over the world. More than 350 million tons of rice are harvested and processed annually. As a result of this process, a large amount of rice straw is formed. Rice straw contains about 50–60% cellulose and 15–20% lignin.

The aim of the study was to develop a method for the isolation and carboxymethylation of powdered cellulose-containing products obtained from rice straw using microwave radiation.

## 2. Materials and Methods

### 2.1. Materials

NaOH, pure, for analysis, impurities not more than 1.0% by weight (JSC “Bashkir Soda Company”, Sterlitamak, Russia), isopropyl alcohol chemically, pure, impurities not more than 0.001% by weight (JSC “ECOS-1”, Moscow, Russia), monochloroacetic acid, chemically pure, impurities not more than 0.001% by weight (Nouryon, Delfzijl, The Netherlands), acetic acid, chemically pure, glacial (JSC “ECOS-1”, Moscow, Russia), and ethanol, chemically pure (JSC “Novokuibyshevsk Petrochemical Company”, Novokuibyshevsk, Russia) are used in the study.

### 2.2. Extraction of Cellulose from Rice Straw

Rice straw samples grown in the Republic of Kazakhstan (Kyzylorda region, Akmarzhan variety) were used for the study. The initial sample of rice straw, according to the analysis data, contained 49.7% α-cellulose, 17.2% lignin, and 3.5% ash.

**Figure 1:**
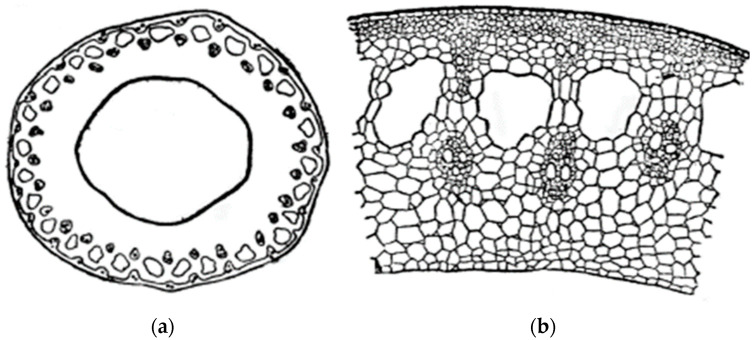
Cross-section of rice straw: (**a**)—low magnification, (**b**)—high magnification.

Numerous studies have shown that the outer hydrophobic layer of the rice stem (outer covering tissue or upper epidermis) (Figure 1) acts as a barrier against the penetration of chemical agents into the lignin–carbohydrate matrix, making it difficult to extract other components. To destroy it, the method of alkaline pulping is most often used.

In order to extract cellulose from rice straw, in the first step, rice straw was cleaned of mechanical impurities and crushed in a crusher to 1–2 cm. The dried rice straw (50 g) was then pounded and stirred to break down the outer cover and remove the main part of the lignin and mineral component for 6 h at 100 °C in 1 L of 1% aqueous NaOH solution. Then the precipitate was separated by vacuum filtration and washed on the filter with distilled water to neutral pH. It was dried in a heating cabinet at 100 °C for 2 h to constant weight. The obtained product was fixed with 89.2% cellulose and 6.7% lignin.

### 2.3. Cellulose Carboxymethylation

**Figure 2:**
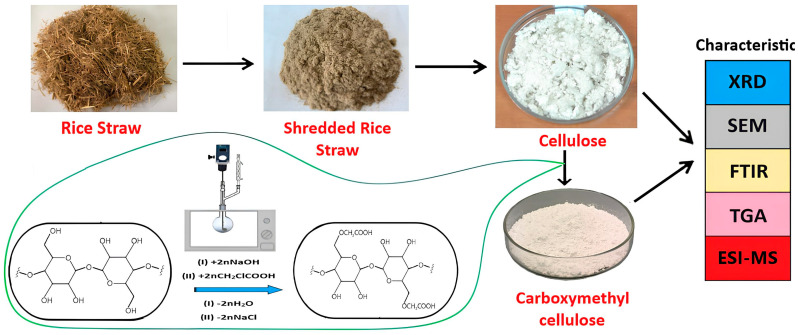
General scheme of lignocellulose carboxymethylation.

The process of carboxymethylation of the obtained lignocellulose was carried out in a microwave oven (900 W). Carboxymethylation is a two-step process (Figure 2). In the first step, the reaction mass, consisting of lignocellulose (5 g) and NaOH (4.2 g) in isopropyl alcohol (50 mL), was activated by exposure to MWR with a power of 350 W for 90 s, as recommended in [48]. At this stage, sodium hydroxide reacts with cellulose, removing hydrogen from the hydroxyl groups at certain positions [49]. The average volume temperature was no more than 75 °C. Monochloroacetic acid (MCA) (6.9 g) was added to the activated reaction mass in the second step and the process was continued under the same conditions (350 W for 90 s).

Then the precipitate was separated on a Buechner funnel and washed with 70% aqueous ethanol solution. A few drops of acetic acid were added to neutralize the alkali. The obtained product was filtered on a vacuum filter and then air-dried. A general illustration of the work is presented in Figure 2.

### 2.4. Measurements

#### 2.4.1. Determination of α-Cellulose and Lignin Content

To determine the mass fraction of α-cellulose, the sample from rice straw, obtained by alkaline hydrolysis with a 17.5% sodium hydroxide solution, was used according to the method used in [50]. The treatment process continued for 30 min at room temperature, after which the precipitate was filtered on a vacuum filter, washed with distilled water, and dried in a heating cabinet at 105–135 °C to constant weight. The weight of the sample was determined on an analytical balance with readability up to 0.0002 g. The lignin content in the sample of rice straw obtained by alkaline hydrolysis was determined according to the method used in [51].

#### 2.4.2. Scanning Electron Microscope (SEM)

The morphology of the isolated lignocellulose was examined on a scanning electron microscope (SEM) JSM-6510 LV (Jeol, Akishima, Japan) in the secondary electron mode at an accelerating voltage of 1.0–1.5 kV.

#### 2.4.3. X-Ray Diffraction Analysis

The phase structure of the samples after alkaline pulping was studied using an X-ray Diffractometer Bruker D5005 (Bruker™, Billerica, MA, USA) with CuKα radiation (λ = 1.5418 Å, power 1.6 kW, 2θ = 5 to 65° at a scanning rate of 4 min and a temperature of 25 °C). The shooting was carried out using the “by-light” scheme in the “diagram recording” mode. For this purpose, tablets were prepared by pressing cellulose at a pressure of 4 MPa. The crystallinity index (*I_cr_*) of the cellulose samples was calculated according to the Segal method [52] by the ratio of the difference between the intensity of the reflex 002 (*I*_002_) and the scattering intensity (*I_A_*) at 18° (at diffraction angles of 2θ) to the total intensity of the reflex 002 (*I*_002_):

$$I_{cr}=\frac{I_{002}-I_{A}}{I_{002}}$$

where *I*_002_ is the intensity of X-ray diffraction at 2θ = 21.7°.

#### 2.4.4. Fourier-Transform Infrared Spectroscopy

Functional group analysis of the powdered lignocellulose from rice straw and its carboxymethylation product was carried out using the IR-Fourier spectrometer «Nicolet iS10» (Thermo Fisher Scientific, Waltham, MA, USA) [53]. The measurements were carried out in the range from 600 to 4000 cm^−1^ and the spectrum resolution was 2 cm^−1^.

#### 2.4.5. Thermogravimetric Analysis (TGA)

The thermal stability of the obtained samples was analyzed using thermogravimetric analysis (TGA) STA 6000 (PerkinElmer, Waltham, MA, USA) by the sample weight loss (%wt.) under heating (heating rate 5 °C/min) in the temperature range of 30–500 °C in a nitrogen atmosphere.

#### 2.4.6. Mass Spectrometry

Ionization mass spectra were obtained by electrospray ionization (ESI) on an AmazonX mass spectrometer (Bruker Daltonik GmbH, Bremen, Germany) with an ion trap. Positive ions were detected in the *m*/*z* range from 70 to 2800. The capillary voltage was 4500 V, the capillary outlet voltage was 140 V, and the drying gas was nitrogen (250 °C, 8 L/min). A solution of methanol/water composition (70:30) with a flow rate of 0.2 mL/min was used as an eluent (chromatograph Agilent 1260, Santa Clara, CA, USA). The sample was injected into the flow through a Rheodyne 7725 injector (Rheodyne, Rohnert Park, CA, USA). TrapControl software (Bruker Daltonik GmbH, version 7.0) was used to control the mass spectrometer and collect data. The data were processed using the DataAnalysis 4.0 program (Bruker Daltonik GmbH, Bremen, Germany).

## 3. Results and Discussion

The sodium hydroxide solution treatment method was selected to extract cellulose from rice straw. This is due to the fact that NaOH, being a strong base, dissolves hemicellulose well. Alkaline treatment leads to the release of minerals, causing swelling of the fibers and partial loosening of the layer between the densely packed fibers of the outer layer of straw. In addition, alkaline treatment removes lignin without destroying carbohydrates and increases the porosity and surface area of the cellulose [54,55]. As a result of alkaline hydrolysis, the cellulose content increased from 49.7% to 89.2%; the content of lignin decreased from 17.2% to 6.7%.

**Figure 3:**
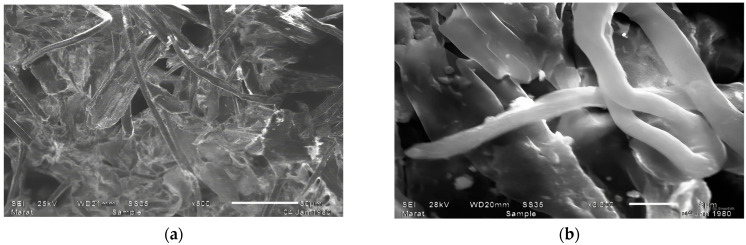
Microphotographs of the lignocellulose after treatment with sodium hydroxide Magnification: (**a**)—x500, (**b**)—x3300.

Microphotographs of the lignocellulose obtained after the removal of mineral components, resins, and part of lignin are shown in Figure 3. The images show a significant change in the structural integrity of the lignocellulose fibers. After mineral removal, the fibers exhibit improved accessibility and a more porous structure that allows for better interaction with chemical agents. The increase in surface area indicates that the dissolution of the mineral components facilitates the identification of cellulose and hemicellulose.

**Figure 4:**
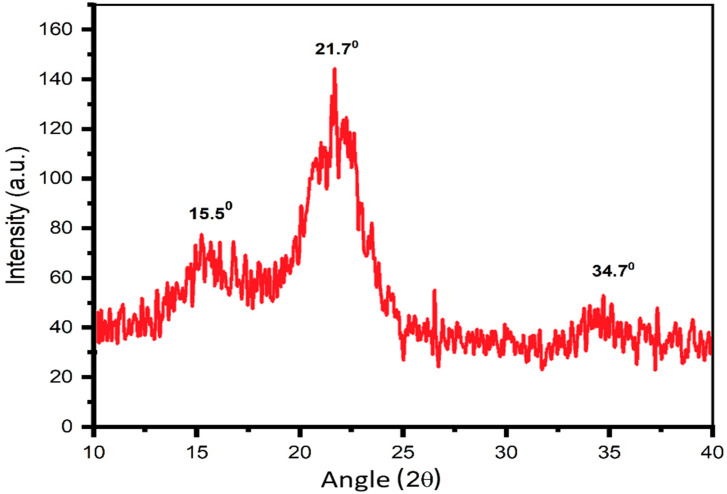
X-ray scattering intensity curves of the cellulose sample after treatment with sodium hydroxide.

Three diffraction peaks were recorded on the X-ray diffraction pattern of the lignocellulose subjected to alkaline treatment (Figure 4): 2θ = 15.50(78), 21.70(145), 34.70(52). The positions of the main reflexes on the X-ray diffraction pattern of the sample are in the region characteristic of the polymorphic modification of cellulose Iβ and correspond to the peaks indicated in [56,57]. The results of the Segal crystallinity index calculations showed that the degree of crystallinity is 65%.

Calculation of the crystallinity index was determined by IR spectroscopy according to the ratio of optical densities of bands D_1370_/D_2900_ [58]. The values of the degree of structure ordering of the cellulose samples calculated from IR spectroscopy data are shown in Table 1.

The 1370 cm^−1^ band characterizes the deformation vibrations of the C-H and CH_2_ bonds. The bands at 2974 and 2887 cm^−1^ characterize the asymmetric and symmetric stretching vibrations of methylene groups, respectively. The band at 2900 cm^−1^ (υC-H) is used as a comparison [59].

The obtained lignocellulose had the stretching vibration band at 895 cm^−1^ associated with deformation vibrations of the C_1_-H bond in the amorphous regions of cellulose [60]. The absorption band in the IR spectra of the obtained lignocellulose samples in the region of 3000 to 3700 cm^−1^ with a maximum at 3326 cm^−1^ characterizes the stretching vibrations of hydroxyl groups included in the hydrogen bond.

The high content of α-cellulose in the obtained sample makes it suitable for further modification. Carboxymethylation was carried out according to the method described above under MWR activation conditions.

**Figure 5:**
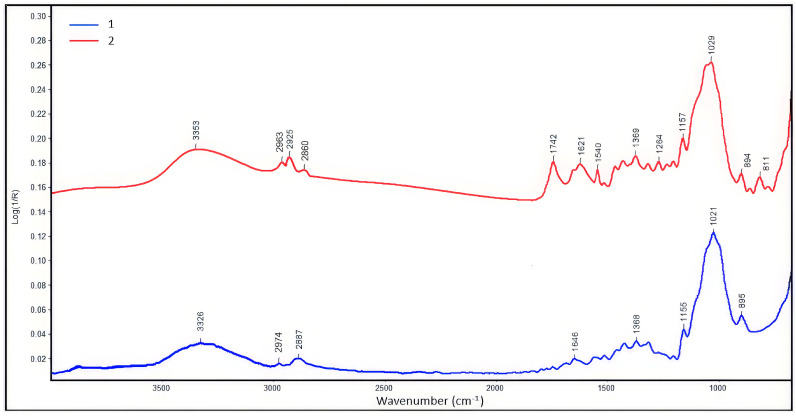
IR spectra (total internal reflection mode): 1—powdered lignocellulose after alkaline pulping, 2—lignocellulose carboxymethylation product.

Functional group analysis of the lignocellulose carboxymethylation product was carried out using IR spectroscopy (Figure 5). Carboxymethylation of lignocellulose led to the appearance of the stretching vibration band characteristic of the carboxyl group >C=O with a peak maximum of 1742 cm^−1^, a vibration band νa of the simple ether bond (C-O-C) in the region of 1028 cm^−1^.

In the product of the carboxymethylation of lignocellulose, a shift of the band associated with changes in the pyranose ring and deformation changes in C_1_-H in amorphous regions to the region of 893 cm^−1^ was recorded (the original sample had the band of stretching vibrations of C_1_-H with a maximum at 895 cm^−1^). The stretching vibration band at 900 cm^−1^ is called the amorphous band because it changes intensity as a result of mechanical or chemical modification of the polymer.

At carboxymethylation of lignocellulose, the wide absorption band of stretching vibrations of O-H bonds with a maximum at 3253 cm^−1^, involved in the formation of inter- and intramolecular hydrogen bonds, was recorded.

The bands at 2860, 2925, and 2963 cm^−1^ characterize the stretching vibrations of methylene groups, respectively. According to Liang and Marchessalt [61], the shape of the peak of methylene groups in this region and its cleavage may be determined by the existence of rotary isomers due to turnings or rotation of -CH_2_OH groups around C5–C6 bonds, as well as the presence of strong bonds between cellulose and its companion—lignin. The absorption bands in the IR spectra of the samples are presented in Table 2.

**Figure 6:**
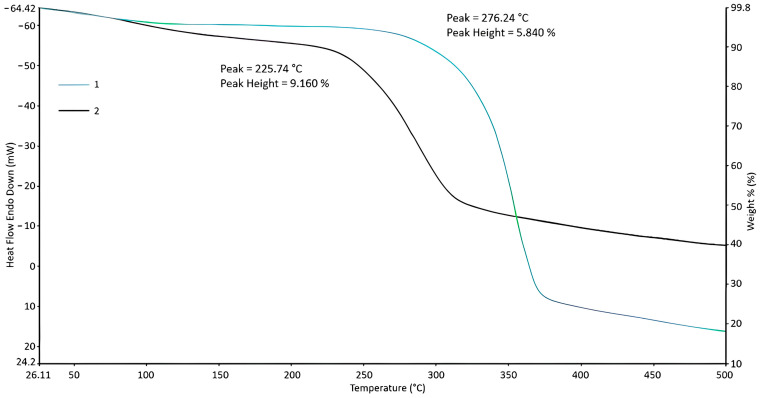
TGA curves: 1—powdered lignocellulose after alkaline pulping, 2—lignocellulose carboxymethylation product.

The thermogravimetry (TG) method was used to evaluate the thermal stability of the obtained products. The results are presented in Figure 6. The study showed that when the powdered lignocellulose obtained from rice was heated to 272 °C, a weight loss of about 6% of the original weight of the sample was observed. This loss is associated with the release of physically bound water, confirming the importance of moisture content in the structure of lignocellulose. Further, during the thermal decomposition of the lignocellulose, weight transfer starts at 276 °C, reaching a maximum at 390 °C, where the total weight reduction is about 71%. This significant level of weight loss in this temperature range indicates the beginning of degradation processes, during which chemical bonds are broken and organic components are converted into volatile products. After 390 °C, there is a period between 390 and 500 °C characterized by a slower decrease in weight, where the loss is about 5%. Carbonization processes continue at this stage. The residue at 500 °C that remains after the completion of thermal processes is 18%. This indicates that a significant part of the sample is degraded, but some of the substance is retained as a stable carbonized residue. As for the carboxymethylated lignocellulose, it is observed during thermogravimetric analysis that it loses about 9% of physically bound water. In contrast to the powdered lignocellulose, the carboxymethylated form shows a significantly more pronounced weight loss at the rapid stage of decomposition, wherein the temperature range 225–355 °C, the loss is about 45%. This indicates that the modification of cellulose increases its reactivity and makes it more susceptible to thermal decomposition at lower temperatures. Thereafter, in the range of 355–500 °C, the carboxymethylated lignocellulose loses about 6% of its weight, which also indicates ongoing thermal degradation processes. The residue at 500 °C, in this case 40% of the initial weight, explains the difference in thermal stability between unmodified and modified forms of lignocellulose. These results emphasize the importance of carboxymethylation for changing the thermal properties of lignocellulose and reveal new perspectives for its application in various fields. The results obtained correlate with the results presented in [62].

**Figure 7:**
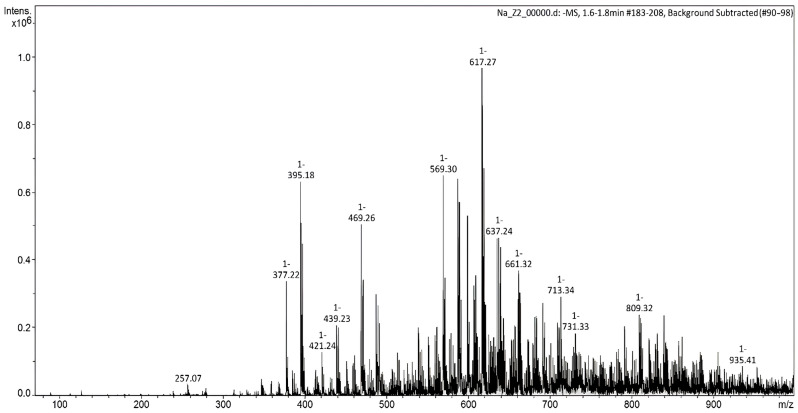
Mass spectrum (ESI-MS) of the lignocellulose carboxymethylation product.

The molecular weight of the carboxymethylation product was determined by mass spectrometry (Figure 7). The mass spectrum shows several intense bands corresponding to the monomeric units of cellulose and its carboxylated form, *m*/*z* (*I_rel_*, %): 469.26 [M–H]^−^.

## 4. Conclusions

This study shows that rice straw is a very valuable source of raw material for the production of cellulose and its chemically modified derivatives. This is particularly important because of the high availability of rice straw as an agricultural by-product. The cellulose extracted by alkaline treatment shows a high degree of purity, consisting of 89.2% α-cellulose and only 6.7% lignin. This purity level is critical for various applications, including food, pharmaceuticals, and biocomposites, where high-quality cellulose is important. In addition, the structural characteristic of the extracted cellulose shows that it has a polymorphic modification known as Iβ. This was confirmed by X-ray diffraction analysis, which showed three distinct diffraction peaks at 2θ values of 15.50°, 21.70°, and 34.70°. The presence of the Iβ polymorphism is important because it is associated with increased crystallinity and mechanical strength, making it suitable for a number of industrial applications.

Another important property of the extracted cellulose is its thermal stability, which is maintained up to 276 °C. This thermal stability is advantageous for applications involving high processing temperatures, ensuring that the cellulose maintains its integrity and performance characteristics under thermal stress.

Moreover, the extracted cellulose can be effectively used in the carboxymethylation process, which is significantly enhanced by microwave radiation. This innovative approach not only accelerates the reaction time but also increases the efficiency of the carboxymethylation process. During this process, a specific stretching vibration band characteristic of the carboxyl group (>C=O) was identified by infrared spectroscopy, with a peak maximum observed at 1742 cm^−1^. This indicates successful modification of the cellulose, which increases its functionality.

In particular, the obtained carboxymethylated lignocellulose exhibits thermal stability up to 225 °C, indicating that the modification process retains its significant thermal stability. This enhanced stability, combined with the new functional groups introduced by the carboxymethylation process, opens up new opportunities for the application of carboxymethylated lignocellulose in various fields, including food supplements, thickeners, and biopolymer formulations.

## Figures and Tables

**Table 1:** The values of the degree of structure ordering of the cellulose samples calculated from IR spectroscopy data.

Sample Number	IR-Fourier Spectroscopy		
	I1	I2	I3
	D_900_/D_2900_	D_1375_/D_2900_	D_1430_/D_2900_
1 *	2.81	2.76	3.21
2 **	2.67	1.83	2.33

* 1—powdered lignocellulose after alkaline pulping; ** 2—lignocellulose carboxymethylation product.

**Table 2:** Absorption bands in the IR spectra of the samples.

Functional Groups, cm^−1^	Initial Lignocellulose	Lignocellulose Carboxymethylation Product
ν(OH), s.	3326	3352
ν(CH_2_), m.	2887, 2974	2860, 2925, 2963
C(O)O	-	1741
δ(HOH), m.	1647	1620
δ(CH_2_OH) + δ(CH), sh.	1422	1427
δ(CH) + γ(CH_2_), m.	1367	1370
δ(OH) + δ(CH_2_), w.	1155	1157
ν(COC)-мocтик, s.	1021	1028
δ(C1H), sh.	895	893

Note. Absorption bands: s.—strong, m.—medium, w.—weak, sh.—shoulder, ν—stretching vibrations, δ—deformation plane vibrations, γ—deformation out-of-plane vibrations.

## Data Availability

Data are contained within the article.
